# Harmonizing adapted interventions across contexts: lessons from harmonizing the World Health Organization’s Caregiver Skills Training in Ethiopia and Kenya

**DOI:** 10.1093/tbm/ibag003

**Published:** 2026-04-09

**Authors:** Melissa Washington-Nortey, Eva Mwangome, Mekdes Demissie, Vibian Angwenyi, Victoria Lewa, Eunice Ombech, Yuan Yao, Tigist Eshetu, Rehana Abdurahman, Fikirte Girma, Ruth Tsigebrhan, WHO CST Team, Laura Pacione, Erica Salomone, Chiara Servili, Amina Abubakar, Charles R Newton, Rosa A Hoekstra

**Affiliations:** Department of Psychology, Institute of Psychology, Psychiatry and Neuroscience (IoPPN), King’s College London, London, United Kingdom; Neuroscience Group KEMRI-Wellcome Trust Research Programme, Centre for Geographic Medicine Research (Coast), Kilifi, Kenya; Department of Psychiatry, College of Health and Medical Sciences, Haramaya University, Dire Dawa, Ethiopia; Centre for Innovative Drug Development and Therapeutic Trials for Africa, College of Health Sciences, Addis Ababa University, Addis Ababa, Ethiopia; Institute for Human Development, Aga Khan University, Nairobi, Kenya; Neuroscience Group KEMRI-Wellcome Trust Research Programme, Centre for Geographic Medicine Research (Coast), Kilifi, Kenya; Institute for Human Development, Aga Khan University, Nairobi, Kenya; Neuroscience Program, The University of Western Ontario, London, Ontario, Canada; Centre for Innovative Drug Development and Therapeutic Trials for Africa, College of Health Sciences, Addis Ababa University, Addis Ababa, Ethiopia; Department of Psychiatry (School of Medicine), College of Health Sciences, Addis Ababa University, Addis Ababa, Ethiopia; Department of Psychiatry (School of Medicine), College of Health Sciences, Addis Ababa University, Addis Ababa, Ethiopia; Department of Psychiatry (School of Medicine), College of Health Sciences, Addis Ababa University, Addis Ababa, Ethiopia; Department of Psychiatry, WHO Collaborating Centre in Mental Health Research and Capacity-Building, School of Medicine, College of Health Sciences, Addis Ababa University, Addis Ababa, Ethiopia; Department of Mental Health, Brain Health and Substance Use, World Health Organization, Geneva, Switzerland; Department of Mental Health, Brain Health and Substance Use, World Health Organization, Geneva, Switzerland; Division of Child and Youth Mental Health, Department of Psychiatry, University of Toronto, Toronto, Canada; University of Milano-Bicocca, Milan, Italy; Department of Mental Health, Brain Health and Substance Use, World Health Organization, Geneva, Switzerland; Neuroscience Group KEMRI-Wellcome Trust Research Programme, Centre for Geographic Medicine Research (Coast), Kilifi, Kenya; Institute for Human Development, Aga Khan University, Nairobi, Kenya; Neuroscience Group KEMRI-Wellcome Trust Research Programme, Centre for Geographic Medicine Research (Coast), Kilifi, Kenya; Department of Psychiatry, University of Oxford, Oxford, United Kingdom; Department of Psychology, Institute of Psychology, Psychiatry and Neuroscience (IoPPN), King’s College London, London, United Kingdom

**Keywords:** Psychosocial Intervention, Contextual Adaptation, Clinical Trials, Developmental Disabilities, Implementation Science, Low-and Middle-Income Countries

## Abstract

**Background:**

Despite the increasing evidence on the importance of contextual adaptation of interventions, there is limited understanding of how intervention adaptations made for one context can inform adaptation and implementation efforts in others or how to harmonize adapted interventions for use across multiple contexts.

**Purpose:**

This paper outlines the process used to further adapt and harmonize previous Kenyan and Ethiopian cultural and contextual adaptations of the World Health Organization’s Caregiver Skills Training intervention as a case study to propose a framework for similar efforts. It also outlines key lessons learned.

**Results:**

The Compare, Decide, Develop drafts, and Test and train (CoDDaT) framework is a four-phased, stepwise approach to intervention adaptation and harmonization that involves: (i) comparing available intervention materials and measures across contexts, (ii) deciding in collaboration with key stakeholders on adaptations needed, (iii) developing drafts of harmonized intervention materials and measures, and (iv) testing harmonized intervention materials and measures and training staff. It complements existing implementation frameworks by describing the preparatory phase ahead of implementing an adapted or harmonized project. Critical lessons learned included the importance of accurate documentation, the team’s phase-specific size and composition, time management, and the opportunities for network growth and skills development offered by the harmonization process.

**Conclusions:**

We demonstrate how lessons from context-specific adaptations can be applied cross-culturally, while incorporating critical revisions and preserving core intervention components. CoDDaT may inform efforts to adapt and implement interventions to promote health behavior change across contexts and advance the field by challenging interventionists to move beyond single-context-focused adaptations and consider applying adapted interventions to other similar contexts, harmonizing, or refining adapted interventions for more extensive use.

## Graphical abstract

**Graphical Abstract:**
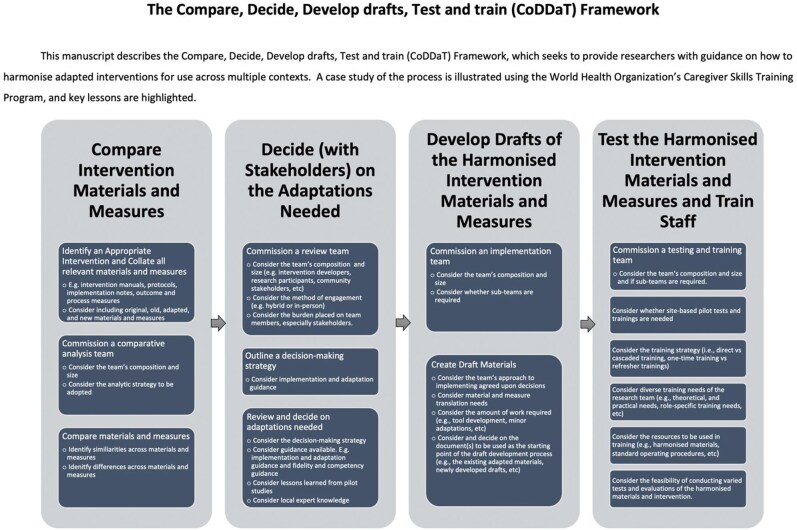

Implications **Practice:** Teams involved in adaptation exercises must maintain accurate records throughout the process to support future intervention harmonization efforts.

**Policy:** Grant funding agencies should permit the inclusion of a development phase in projects that include a harmonization component and develop data repositories to ensure effective implementation and maximize the impact of behavior change interventions.

**Research:** In lieu of developing newly adapted interventions, research teams should consider reusing and harmonizing adapted interventions for projects being implemented in contexts like those in which the adapted interventions were first used.

## Introduction

Over 80% of people with mental illness and ∼95% of children with developmental disabilities live in low- and middle-income countries (LMICs) [1, 2]. Despite this burden, across varied disciplines, including psychology, public health, and mental health, there is limited evidence of interventions developed in and for people in these contexts [3, 4]. Interventions transferred from higher-resourced contexts to these regions often fail or achieve limited success because they do not account for important cultural and contextual factors that would engender their success in these areas [5, 6].

Several frameworks, including the United Kingdom’s Medical Research Council (MRC) and National Institute for Health and care Research’s (NIHR) framework for complex interventions and the Exploration, Preparation, Implementation, and Sustainment (EPIS) framework, developed by researchers in the United States, have been drafted to guide the development, implementation, and evaluation of interventions in varied contexts. The MRC–NIHR framework conceptualizes complex interventions as “events in systems” that interact with contextual factors within the intervention’s setting to produce an outcome [7]. It outlines four intervention research phases comprising the development or identification of an intervention, conducting feasibility assessments, evaluating, and implementing interventions. Further, it recommends that throughout intervention development and evaluation, core elements are considered, including context, stakeholder input, the program theory, and economic considerations. Like the MRC–NIHR framework, the EPIS framework also describes a four-phased process but specifically describes an implementation trajectory progressing through the process of: *exploring* options available to address an identified concern/challenge, *preparing* the intervention materials and preparing all stakeholders including the intervention delivery team and intervention recipients for the intervention, *implementing* the intervention, and *sustaining* the intervention and/or the desired positive impacts for an extended period [8]. Within each phase, it highlights the role of context by describing the contribution of internal and external factors in shaping the final intervention outcome(s). These and other frameworks have improved the quality and effectiveness of interventions by encouraging greater consideration of relevant factors and elucidating the mechanisms underlying outcomes.

Recent work has focused on the systematic, proactive adaptation of interventions to increase their fit, acceptability, and appropriateness for specific contexts [9–11]. Adaptation efforts are premised on two essential principles: the adoption of effective interventions and the contribution of contexts to implementation outcomes. Specifically, adaptation exercises seek to prudently implement effective interventions or components of effective interventions in novel contexts, instead of developing new interventions for each context [12]. Yet, recognizing that effective interventions often fail in new contexts because they overlook relevant contextual factors, it seeks to increase their chances of success by modifying existing components or incorporating additional ones to enhance the intervention’s fit. Aiming to increase the quality and reporting of adapted interventions, several adaptation frameworks have been developed [12–15]. There is also growing evidence base showing the effectiveness of adapted interventions over nonadapted alternatives [3, 14, 16, 17].

The critical contributions of contexts to the successful implementation of original and adapted interventions have also prompted the development of implementation science frameworks within the field of implementation research: “the scientific inquiry into questions concerning implementation [18].” These frameworks guide researchers in delineating determinants (i.e. barriers and facilitators), including context-specific determinants, that may influence the outcomes of implementation efforts and interventions. Examples of these implementation frameworks include the Consolidated Framework of Implementation Research (CFIR) [19] and the Context and Implementation of Complex Interventions framework [20].

Advances in intervention development, adaptation, and implementation research have improved the quality and reporting of interventions. Yet, they fail to address specific gaps and emerging challenges adequately. To illustrate, just as broad overall contextual similarities can facilitate the implementation of original interventions in other contexts with minimal change, adapted interventions may also be implemented in alternative yet similar contexts with limited need for additional changes. Interventions adapted to specific contexts that share similar characteristics may also be harmonized to allow cross learning. Thus, from a methodological standpoint, it is crucial to develop intervention methods that facilitate the evaluation of an adapted intervention’s value for a similar context, as well as comparisons across adapted versions of the same intervention. This will also permit the drawing of meaningful conclusions on the intervention’s acceptability, ­feasibility, and efficacy across various contexts.

However, most guidance currently available for adapting ­interventions, such as the ADAPT framework, which outlines the adaptation process [12], focus on implementation within a single context. There is a dearth of literature available to inform researchers on how interventions adapted for a specific context can be adapted and harmonized for use in other similar cross-cultural, linguistic, and rural/urban contexts. Additionally, we have not identified any literature providing guidance on (i) how to assess the integrity of an intervention adapted for alternative use, (ii) how to determine the usefulness of specific adaptations made in the adapted version for a new context that shares contextual similarities, or (iii) how to determine and decide on additional adaptations that may be needed in the new context.

We operationally define intervention harmonization as a systematic attempt to assess the similarities and differences between intervention materials, content, protocols, and strategies used in different contexts to streamline and standardize them for use across multiple contexts, in case of contextual similarities. We conceptualize harmonization as distinct from adaptation but note that further adaptation may become necessary during the harmonization process. A harmonized intervention, including its manuals and recommended protocol or procedures, is the product of the harmonization effort.

The approach can be used to (i) apply context-specific adaptations (e.g. Kenyan versions) of an intervention to another specific context with shared characteristics (e.g. another country such as Tanzania), (ii) integrate and consolidate context-specific adaptations to capitalize on shared learning and to create a version for a broader context (e.g. an East African version) that can subsequently benefit other similar countries, or (iii) create a version for a broader context (e.g. Latin American version) that would subsequently require further adaptation tweaks for specific contexts to enhance fit (e.g. for a specific Latin American country such as Uruguay). Harmonizing previously adapted interventions for broader use in similar but alternative contexts can reduce the need for extensive preimplementation adaptation, increase efficiency in resource use, promote shared learning across contexts and research groups, promote the implementation of more complex intervention studies with larger sample sizes, increase the generalizability of findings, and increase research impact.

Shared characteristics within and across cultures can facilitate the successful transfer of interventions, including health behavior change interventions, with minimal need for further adaptation. For example, the Problem Management Plus intervention [21], a WHO-developed low-intensity psychosocial intervention that aims to reduce the symptoms and practical challenges associated with common mental health problems like anxiety, distress, and depression, has been successfully implemented in countries such as Colombia, Ethiopia, Kenya, Turkey, and the United Kingdom [22]. Its success is partly attributable to its explicit development for use in LMICs, minimizing the need for extensive adaptation across LMIC settings. Yet, even when contexts share important core characteristics, other critical, unique characteristics, and nuances may exist that can impact an intervention’s success. Therefore, following a systematic approach to harmonizing or selecting adapted interventions for use in alternative contexts can help delineate these unique factors and inform any additional nuanced adaptations needed to increase the intervention’s prospects in the new setting.

This paper outlines the process of adapting and harmonizing the World Health Organization’s Caregiver Skills Training (WHO CST) intervention for a cross-cultural multicountry clinical trial in Kenya and Ethiopia [23], showing an example of harmonization that incorporates adaptation and involves consolidating two adapted versions of an intervention. By so doing, it also presents an example of an adaptation exercise from LMICs, from which limited evidence exists, to inform the field [11, 24]. Additionally, it highlights key lessons learned from the process.

## The WHO CST for families of children with developmental delays or disabilities

The WHO CST is a caregiver-mediated intervention that equips caregivers of children with developmental disabilities with skills to promote their children’s engagement in activities, social communication, daily life skills, and behavior management [25, 26]. It also seeks to improve caregivers’ well-being by promoting health-related behavior changes in self-care practices, encouraging engagement with other caregivers facing similar difficulties, and teaching stress reduction strategies. The intervention has undergone adaptation and field tests in over 30 countries [25, 27], including Kenya and Ethiopia, where, in line with WHO recommendations, field test versions were contextually adapted and pilot-tested [28, 29]. Findings from the initial multicountry pilot-tests informed the WHO CST’s revision and final versions were published in 2022 on the WHO website [25]. In 2020, in preparation for conducting a comprehensive Randomized Controlled Clinical Trial of the WHO CST in Kenya and Ethiopia, the SPARK (SuPporting African communities to increase the Resilience and mental health of Kids with developmental disorders and their caregivers) research team compared and harmonized the previously adapted versions of the WHO CST field test versions used in their respective country-level pilots with the newly published final versions. The SPARK project is an international research collaboration between the following institutions in the United Kingdom, Kenya, and Ethiopia: King’s College London—United Kingdom, the University of Oxford—United Kingdom, Aga Khan University—Kenya, the Kenya Medical Research Institute/Wellcome Trust Research Programme—Kenya and Addis Ababa University—Ethiopia. It is implemented in collaboration with a parent led organization, Nia Foundation-Joy Autism Centre and a community-based organization, Kuhenza for Children. It is a two-phased study specifically aimed at (i) promoting the community-based identification of children with developmental disabilities using the Community Identification of Developmental Disabilities tool, developed by the team, and (ii) investigating the effectiveness and cost-effectiveness of the WHO CST program through a cluster randomized clinical trial. Details of the project, which is being implemented in a rural and urban site in each country, are outlined in its published protocol paper [23]. This manuscript’s objectives are:

1. To describe a framework derived from steps taken to adapt and harmonize previously adapted versions of the WHO CST intervention and the final published versions for use in the cross-country Randomized Controlled Trial.
2. To outline and discuss critical lessons learned.

## Method and results

**Figure 1:**
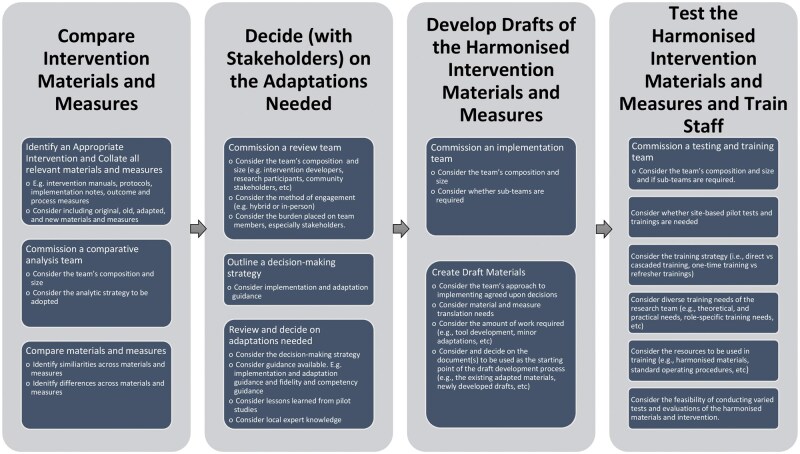
The Compare, Decide, Develop drafts, Test and train (CoDDaT) framework.

We developed the Compare, Decide, Develop drafts, and Test and train (CoDDaT) framework as a four-phased stepwise approach to adapt and harmonize interventions. Specifically, CoDDaT involves: (i) comparing available intervention materials and measures across contexts, (ii) deciding in collaboration with key stakeholders on adaptations needed, (iii) developing drafts of harmonized intervention materials and measures, and (iv) testing harmonized intervention materials and measures and training staff. See Fig. 1 for an overview of the CoDDaT framework. We consider this framework as complementary to theoretical frameworks that recommend careful consideration during the planning and preparatory phases of intervention development, as it addresses specific gaps linked to the preparatory phase of repurposing or harmonizing adapted interventions. As the EPIS framework explicitly includes a preparatory phase, we use it to illustrate the framework. The exploratory, implementation, and sustainment phases, as described in the EPIS framework, remain applicable to this work and should be referenced by teams. We explain each phase of the CoDDaT framework (Fig. 1) using the WHO CST intervention as a case study.

### Phase 1: Compare available intervention materials and measures

This first step involves identifying the appropriate intervention for a given context and collating all intervention-related materials needed for successful implementation. Unlike an exercise solely aimed at adapting an intervention for a context that requires selecting an original intervention to address an identified need [12], this stage in a harmonization exercise requires selecting an intervention that targets the identified problem and has previously been adapted and implemented in contexts similar to the target study region or population. Ideally, there should be implementation evidence in the target study region(s) and populations. However, in the absence of this, adaptation evidence from similar regions or populations may suffice. Once an appropriate adapted intervention has been selected, teams must collate all relevant intervention materials and compare materials used in the ­different projects. Below, we describe the SPARK team’s process at this stage.

#### The materials and measures

These refer to all intervention materials, such as manuals—for manualized interventions—protocols, implementation notes, outcome and process measures, etc., that informed or were used in previous studies.

The WHO CST is a manualized intervention comprising nine group sessions and three individualized home visits occurring at the beginning, middle, and end of the group sessions [25, 26]. The intervention is designed to be delivered by trained nonspecialists (i.e. people without specialist professional training or clinical degrees) using group session facilitator and participant guides, home visit guides, and some recommended child and caregiver assessment instruments. The Kenyan and Ethiopian pilot CST materials had previously been adapted and pilot tested in a process comprising: (i) extensive stakeholder engagement on the context’s training needs, the CST materials and content, and considerations for its implementation and delivery; (ii) translation of the CST facilitator and participant guides into the local language; and (iii) a pilot with caregivers of children with developmental disabilities. Detailed accounts of the Ethiopian and Kenyan adaptations can be found in Tekola *et al.* [28] and Abubakar *et al.* [30]; a description of the training of nonspecialists and pilot evaluation of the program delivered by nonspecialists in Ethiopia is provided in Zerihun *et al.* [31]. Consequently, the collated materials for the harmonization exercise included adapted group session facilitator guides, adapted participant guides, adapted home visit guides, varied participant questionnaires, and other process documents like meeting minutes evidencing an audit trail of the adaptations. We also obtained, from the intervention developers, tracked-changed versions of the newly published WHO CST materials incorporating feedback from several global pilot studies and field tests.

#### The comparative analysis team and the comparative process

This refers to the group comparing all materials and measures used in the previous studies and the process of identifying areas of congruence and incongruence across the different study contexts. The strategies employed in this process may be akin to those used when determining an intervention strategy’s suitability within a specific context, as reported in previous studies [32]. However, here, the primary goal is to determine whether the same materials and strategies were used in all contexts where the intervention was previously implemented and to note all similarities and discrepancies. Contextual suitability assessments are conducted in the second phase.

In SPARK, the team conducting the comparative analysis (*n* = 4) comprised nonspecialist facilitators from the CST pilot test and current project coordinators. First, we compared all the adapted CST materials/guides used in the pilot studies, noting all similarities, differences, and adaptations implemented on Excel spreadsheets. Excel tabs dedicated to each resource identified (e.g. intervention manuals) were populated with details of the resource’s components and sections, as applicable. Team members documented whether these components or sections were identical or discrepant across site-specific materials. Where discrepancies or adaptations were noticed, their nature was outlined. Thus, the process generated Excel spreadsheets with tabs for each resource, which guided later discussions in Phase 2. Similarities across teams included the replacement of characters’ names in the original WHO CST intervention guides with local names reflecting their respective contexts. Differences included variations in intervention content and delivery approaches, such as the retention of participant writing assignments with support from available staff in Kenya, and the removal of all such assignments in Ethiopia to accommodate the lower literacy levels of caregiver participants in that context. Shortly after this initial comparative work with the pilot-based materials was completed, we received the newly revised and published WHO CST materials [25] from the intervention developers and engaged in a similar activity documenting differences and similarities between the new WHO CST materials and each country’s CST pilot materials.

Lastly, we compared the types and versions of outcome measures used. As several resources exist on general measure comparison, identification, and adaptation e.g. [33], we do not discuss these here. We focus exclusively on measures that primarily aid the accurate interpretation of intervention effects: measures of intervention fidelity—the extent to which an intervention is delivered as desired—and of intervention delivery competence—the skill-levels of staff engaged in intervention delivery [34]. These included adapted versions of the ENhancing Assessment of Common Therapeutic Factors (ENACT) [35], which assessed competency in delivering the CST group sessions, and the Activity Completion Checklist, a newly developed fidelity instrument measuring the extent to which the CST nonspecialist facilitators implemented all aspects of the CST group session manuals. Both tools were used in the Ethiopian pilots only, and thus, no comparison was necessary.

### Phase 2: Decide in collaboration with key stakeholders on adaptations needed

Like a typical adaptation study, this phase would involve considering aspects of the intervention needing adaptation and the appropriateness of the proposed adaptation for the target population [32]. However, it also specifically involves commissioning a team to review the differences in adaptations implemented across studies based on the comparative analysis results, considering the relevance and value of previously implemented adaptations, as well as agreeing on adaptations to retain and areas requiring further adaptation and harmonization.

#### The review team

These are the people enlisted to review the comparative analysis results and propose appropriate actions. Considerations for membership include intervention developers, research participants, policymakers, invested community representatives, and the research team [32, 36]. Careful thought must be given to their selection, as their diverse perspectives, positions, and interests could greatly enrich or introduce tensions that could impede the process. Our review team, comprising ∼30 individuals, included trained CST nonspecialist facilitators who had delivered the CST sessions to caregivers in their respective country-specific CST pilots, newly trained CST nonspecialist facilitators involved in the SPARK project, specialist clinicians who assessed children suspected of having developmental disabilities, the WHO intervention developers, and researchers who led the CST pilot studies and have remained involved in the program’s revision. While there were no policymakers or caregivers of children with developmental disabilities directly on the review team, input from these important stakeholders was sought through engagement with our country and site-specific project and community advisory boards, who also included parents of children with developmental disabilities and policymakers. The CST nonspecialist facilitators from the country-specific pilots also shared insights from stakeholder engagement and feedback obtained during those projects. This input was carefully considered in the review and decision-making process.

##### Method of engaging the stakeholders

The method of engaging stakeholders depends on factors such as their number, range of expertise, availability, and residential location. Where possible, extended face-to-face meetings that allow for clearer, more nuanced discussions are ideal. Nonetheless, virtual and hybrid meetings offer an acceptable substitute where practical constraints limit in-person meetings. We organized a series of online cross-country harmonization meetings via videoconferencing to discuss findings from the comparative analysis. The online nature of these international cross-site meetings, the volume and often technical nature of the documents to be reviewed, and the use of the English language meant that more marginalized groups and policymakers were not directly included in this step. Instead, out of respect for their time and to maximize our engagement, we distilled relevant topics and issues for their input during dedicated advisory board meetings. Nonetheless, we recognize their exclusion from the main team as a clear limitation of our approach.

##### The decision-making strategy

**Figure 2:**
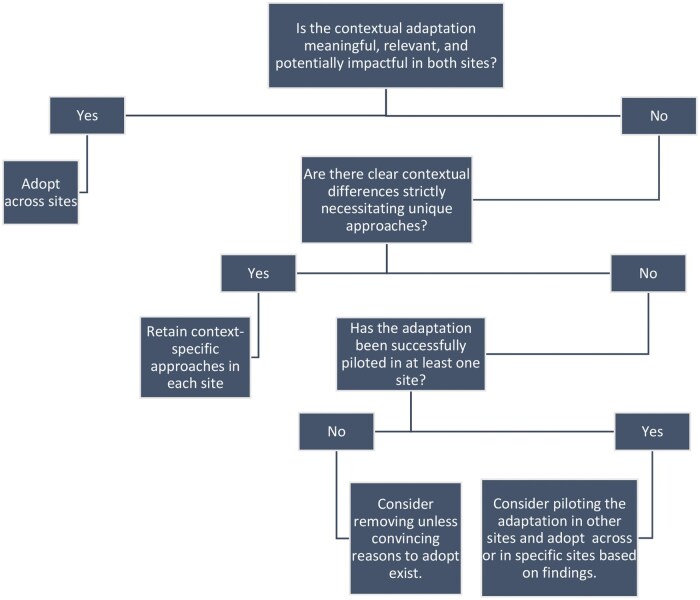
Decision-making strategy for adapting and harmonizing the WHO CST intervention for use in Ethiopia and Kenya.

This considers how decisions are made about harmonizing the intervention materials and whether adaptations are needed. Prior research has distinguished between altering core intervention components, theorized as critical in producing the desired change, and making changes to less critical components, often to increase the intervention’s cultural and contextual appeal [32]. While the risk of intervention failure is higher when core components are altered, researchers also note that it is often challenging to identify these essential core components, and they may differ in contexts with other norms [32, 37, 38]. Using a predetermined decision-making strategy and adaptation guides can help safeguard the adaptation process while ensuring consistency. We used the WHO’s adaptation and implementation guide as an overarching framework [39] and outlined strategies to guide all harmonization and adaptation decisions after noted differences were discussed within the meetings or in further consultations with subject-matter experts, as needed. Final decisions were made through a consensus-based discussion. The decision-making strategy is outlined in Fig. 2.

With the decision-making strategy, we made contextually informed changes based on recommendations in the CST adaptation and implementation guide, lessons from the respective pilot studies, and expert knowledge of the local context from researchers and community stakeholders, to the content and mode of delivering the intervention. Content-changes comprised alterations to the information covered (i.e. the what), whereas changes to the delivery mode affected *how* the intervention was delivered. Examples of content changes included adding a section addressing physical punishment and redistributing some content across sections to achieve greater equity in session volume. Examples of delivery-mode changes were the organization of home visits at central locations in all sites and introducing supports for less literate caregivers to complete session-based writing tasks. The changes sought to improve the intervention’s face validity, achieve greater balance in the content volume across sessions, and address important cultural issues linked to childcare and behavior management in these contexts that could affect the intervention’s perceived relevance and eventual successful implementation.

#### The fidelity and competency measures

The adapted ENACT (competency) and Activity Completion Checklists (fidelity) used in the Ethiopian pilot studies were deemed relevant for the Kenyan context, as they both related specifically to delivering the CST group sessions and assessed aspects that were standardized across the teams. Thus, they were adopted in the Kenyan context. Another fidelity measure, a video-based assessment of caregiver-child interactions, was considered but not used due to logistical and resource constraints. To ensure consistent implementation of supervision across all sites in the much larger SPARK trial, a new supervision record form was developed to record supervision sessions systematically.

### Phase 3: Develop drafts of the harmonized intervention materials and measures

This phase considers all the processes involved in implementing the decisions taken in the previous step such as commissioning implementation teams, outsourcing specific tasks, and updating or developing draft materials based on decisions made in the prior phase.

#### The implementation team

SPARK subteams or hired professionals implemented the decisions. The Ethiopian implementation team comprised project coordinators, CST master trainers and supervisors, and professional translators (*n* = 6 in total), whereas, in Kenya, all changes were implemented in-house by project coordinators, CST supervisors, and nonspecialist facilitators (*n* = 11). Both teams included staff who had participated in the harmonization meetings and could provide helpful contextual information about the recommended changes.

#### Creating the draft materials

The CST materials, published in English, were harmonized and translated into each country’s most popular local language: Amharic in Ethiopia; Swahili in Kenya. In Ethiopia, the SPARK team implemented all changes to the English CST materials in a series of working meetings that methodically progressed through all the materials and the recommended changes. Trained professionals then updated the Amharic CST pilot materials to incorporate changes implemented in the newly updated English versions. Changes to the Kenyan documents were also decided in collaborative working meetings, but in contrast to the Ethiopian approach, the SPARK-Kenya implementation team implemented all agreed-upon changes directly onto the adapted Swahili documents used in the pilot. Resource challenges hindered the team’s ability to create English equivalents of the Kenyan CST materials. The harmonized materials are available on request from the SPARK executive group at SPARK@kcl.ac.uk.

All intervention fidelity and competency measures, except for the supervision record form, were preexisting questionnaires. Only the Activity Completion Checklists required minor changes reflecting the redistributed content across sessions. The supervision record form was drafted in English and not translated, as all supervisors speak English.

### Phase 4: Test the harmonized intervention materials and measures and train staff

This step highlights the process and importance of piloting and evaluating the materials and measures, as well as training staff with the developed drafts. Here also, considerations about the team and strategies are equally valid. It requires research groups to consider the types and extent of evaluations necessary, whether adapted materials should be evaluated independently or within the ongoing study, and whether further adaptation is needed based on the evaluation results [12].

#### The testing and training team

This comprises trainers and trainees. WHO CST intervention developers, Ethiopian CST master trainers, and project and trial coordinators served as the trainers (*n* = 10). Trainees included CST master trainers, supervisors, and nonspecialist facilitators. In Ethiopia, we used a cascaded train-the-trainer model: WHO CST intervention developers trained Ethiopian CST master trainers who had previous CST experience and newly employed CST supervisors. The master trainers and supervisor then trained local nonspecialist facilitators. Language and other logistical challenges prevented the Ethiopian nonspecialist facilitators from receiving direct training from the intervention developers. The cascaded training model was not adopted in Kenya, as there were no Kenyan CST master trainers. Instead, the WHO CST intervention developers jointly trained the supervisors and nonspecialist facilitators.

#### Testing the WHO CST materials and measures and training the intervention delivery team

Material testing and training were conducted concurrently. Some training sessions were organized at a country level, whereas others were joint sessions with trainees from both countries. The CST master trainers, supervisors, and nonspecialist facilitators in both countries were trained in the theoretical and practical aspects of delivering the CST using the harmonized materials, which also served as a preliminary pragmatic test of the material’s suitability and a check for the accuracy of all revisions made.

The WHO CST intervention developers led an initial theoretical and practical training of the Kenyan and Ethiopian teams in half-day online sessions following the training models specified above. Subsequently, both teams practiced their skills by engaging groups of volunteer caregivers of children with developmental disabilities in a pilot run of the full CST intervention. Mirroring real-world implementation timelines and formats, these were conducted in person and lasted ∼12 weeks. The same training models were used to deliver periodic booster training sessions across sites, although some of these comprised full-day in-person workshops depending on the availability of trainers and trainees. Additionally, the CST master trainers and supervisors across sites participated in monthly 1.5-h-long online meetings with the intervention developers to further strengthen their intervention-delivery competencies.

Project and trial coordinators drafted bespoke Standard Operating Procedures: guidelines that ensure consistency in intervention delivery and questionnaire administration [40]. They were used to train the CST master trainers, supervisors, and nonspecialist facilitators administering and using the fidelity and competency measures. Insights from the training informed the revision and finalization of the operating procedures, materials, and measures. For instance, the CST’s procedures recommend conducting individualized home visits in participants’ homes. While desirable, logistical and security-related challenges rendered this unfeasible in most contexts. Therefore, the team transitioned to individualized child–caregiver visits in a convenient but central location. This testing phase also aided in identifying translation revisions needed to enhance clarity and comprehension in the manuals. In addition, the phase allowed for the assessment of specific adaptations made, such as whether changes made to equalize the amount of content per session were successful.

Although our use of the drafted harmonized WHO CST materials and measures during training permitted preliminary checks for comprehensibility, accuracy, and further refinement needs, there remains a need for a more formal, robust evaluation of the harmonized intervention. The effectiveness of the CST intervention will be tested in full in the SPARK trial [23] and in an associated ­implementation science-focused process evaluation investigation exploring contextual factors that influenced the intervention’s implementation [41]. More details on intervention evaluation are presented in the EPIS framework [8].

## Key lessons learned and implications

In this section, we highlight five key lessons learned and their implications to inform similar efforts: (i) the importance of documentation, (ii) the critical role of the team’s composition at each phase, (iii) the value of having predetermined decision-making strategies, (iv) an awareness of the time investment, and (v) the opportunities afforded by the harmonization process.

First, the importance of having accurate, reliable records of all intervention materials cannot be overstated. This work relied heavily on archival records from previous pilot studies and new records from the recent revisions to the WHO CST. The adapted intervention materials from the pilot studies, including the formal and informal correspondence, anchored the comparative analysis and permitted an initial assessment of the scope of work and our resource capacity. The process documents also helped contextualize, clarify, and elucidate specific modifications implemented in each country. Further, including the final published version of the CST materials [25], resulted in an updated version of the intervention that addressed important limitations identified in other pilot and field tests. However, in practice, researchers may not have access to all intervention materials ahead of the comparative analysis stage. This should not unduly delay the process, as it may be possible to integrate new materials later in the comparative work. Obtaining accurate and detailed records can also help research teams assess the intervention’s fit for their contexts [12]. Teams embarking on similar initiatives should strive to obtain all relevant materials and information, including such intervention updates and consider supplementing these materials with interviews of research staff involved in the original studies to capture anecdotal insights omitted from written correspondence. Considering the immense value of clear, accurate documentation in this process, researchers are also encouraged to maintain accurate records while adapting interventions to support future efforts. Researchers and funding bodies may also consider utilizing research repositories such as the Open Science Framework or dedicated platforms where intervention materials can be stored and openly accessed to advance similar initiatives.

Second, the team’s size and composition at each stage of the adaptation and harmonization process are crucial. We found that smaller teams were appropriate at the comparative analysis, ­decision implementation, and testing and training stages. These smaller, sometimes site-specific teams, primarily composed of experienced staff, increased efficiency and consistency during implementation and facilitated the larger team’s subsequent training. In the comparative analysis stage, having a small team of staff experienced in the intervention aided an efficient review and comparison of the materials and questionnaires.

In contrast, the decision-making stage required input from varied stakeholders and a larger, more diverse team. Their diverse perspectives greatly enriched discussions and the quality of decisions made for all intervention components. For instance, the researchers, some of whom are based in these contexts and others from elsewhere, provided insight into the theoretical perspectives and cultural norms, practices, and values that motivated changes to the intervention’s content and mode of delivery. These insights were complemented and sometimes challenged by the perspectives of the CST implementors—master trainers, supervisors, and facilitators—and the WHO intervention developers. The CST implementors helped ensure the adoption of feasible, acceptable strategies, whereas the WHO intervention developers helped steer the team away from making changes to the perceived core components of the intervention. Lastly, we presented some of the major adaptation considerations to a wider group of community stakeholders to ensure the voices of policymakers and caregivers of children with developmental disabilities (i.e. the program’s target beneficiaries) were also heard. These varied perspectives informed content, context, and implementation-based adaptations made to the intervention’s materials and procedures. Employing site-specific teams in the draft development phase also facilitated updating previously used pilot materials, especially since some of these changes were incorporated directly into the existing translated materials. When commissioning teams for each phase, research groups must carefully consider both project needs and the internal and external human and financial resources available to execute tasks. Larger research groups may more easily assign team members to specific phases, but all teams are encouraged to consult with stakeholders outside the research team to obtain diverse perspectives on materials, approaches, and strategies, while ensuring that more marginalized voices are heard throughout the process.

Although our experience was positive, if not well-managed, the differing interests, investments, orientations, and roles in such teams can introduce tensions that could negatively impact operations or outcomes [10, 42]. Our third lesson, the value of having predetermined decision-making strategies, focuses on this challenge. We mitigated such tensions by adopting a range of effective communication strategies, as well as other strategies informed by the research team’s experience and judgment. These included clarifying the project’s objectives at the outset, considering lived experiences, prioritizing field-based perspectives over untested evidence in the extant literature, encouraging the introduction and discussion of divergent views, and adopting consensus-based decision-making approaches throughout the harmonization process. For instance, the CST intervention materials include suggestions for a toy kit comprising several types of toys to elicit different types of play, ranging from simple to more advanced pretend play. However, deciding on the specific kinds of toys to be included in the standardized cross-site kits required much discussion to ensure appropriateness for the intervention and our contexts. We first consulted with the intervention developers to understand the range of suitable locally available toys to ensure they could cater to a variety of developmental levels. Subsequently, we held team-based discussions, led by CST facilitators, in the pilot and current study on the types of toys available in each context that met the recommendations set forth by the intervention developers and on the feasibility of making or purchasing local toys at the scale needed for the intervention. At the end of the process, we generated a standardized list of appropriate toys to purchase and use across all our contexts. Research teams must carefully consider and select team members best suited for each task to guarantee the project’s success. When allocating tasks, teams should also appraise the burden placed on members, especially stakeholders who may have other formal and informal responsibilities, to avoid overburdening them, even if they are compensated for their involvement. Additionally, large, diverse teams comprising members from different geographic regions should consider the power and authority wielded by all team members and adopt strategies to ensure equitable collaboration [43]. Relatedly, creating and utilizing predetermined decision-making strategies during the decision-making phase was beneficial. Efforts to adapt complex, multicomponent interventions involving large teams can spiral out of control if not well-managed, and we found that having these predetermined strategies helped keep our teams on track and focused. While our approach focused primarily on discrepancies for pragmatic reasons, teams may consider discussing all adaptations made to ground further decisions in evidence.

Effective time management and planning was the fourth essential lesson from the harmonization exercise. The harmonization and adaptation process is a project in its own right. The downtime afforded by the COVID-19 pandemic, when travel and fieldwork were halted, proved to be an unexpected gift during the comparative analysis phase. However, the team needed to carefully juggle team members’ responsibilities during all other stages to ensure timely completion. Time considerations may also extend beyond the immediate project team, depending on the nature of the project. For instance, the draft development phase required translating documents into one prevalent local language for each country. This, in some cases, had to be completed by trained professionals external to the project, which can affect a team’s efficiency. Translation-based projects may require further scrutiny as professionals outside the academic discipline may incorrectly translate technical terms and phrases, impacting the quality of the intervention materials. In contrast, our Kenyan CST team found that completing translations in-house increased the team’s knowledge of the intervention’s theoretical underpinnings and may have strengthened their competencies and confidence in delivering the intervention. Guided by the CoDDaT framework, research teams can develop realistic estimates to incorporate into project timelines and better manage expectations. Funders may also consider permitting the inclusion of a development phase in grant proposals for such projects, allowing dedicated time for harmonization and adaptation work.

Lastly, the harmonization exercise was an excellent opportunity to expand our research network and gain new knowledge and skills. Specifically, the integration of two research teams with pilot-based experience with the intervention led to the discovery of complementary studies, other shared interests, and new measures and methods that strengthened operations across all sites. The site-specific teams also gained insight into potentially relevant cultural adaptations that had not previously been considered, resulting in a more robust intervention. Further, the information exchange was perceived to increase implementation feasibility by introducing tested strategies that were more likely to succeed across contexts, given their similarity. This may be particularly critical to the success of technology-based behavioral change interventions in LMICs, given the need to adopt creative strategies to circumvent limitations in the technologies available for implementation.

### Strengths and limitations of the SPARK case study and the CoDDaT framework

The article’s strengths include expanding the field’s focus from single-context adaptations to harmonizing adapted interventions for use in multiple contexts. It also presents an example of a LMIC-based harmonization exercise that addresses the research gap in evidence of adaptations from non-Western industrialized contexts highlighted in other studies [3, 11, 24]. Additionally, it ­describes the process of harmonizing two previously adapted interventions across two countries, which can inform efforts to implement adaptations from one country to another, e.g. implementing an intervention adapted for the Kenyan context in another East African country.

In comparison to existing guidance on intervention adaptation and implementation, the CoDDaT framework may also offer a number of advantages, including facilitating (i) the identification of factors associated with implementation and intervention outcomes, (ii) a more thorough vetting of intervention adaptations, and (iii) the discovery and consideration of essential determinants that may be missed in bespoke single-context adaptation exercises. First, by utilizing implementation frameworks such as the CFIR [19], to identify essential contextual determinants and by systematically applying the steps outlined above, teams can more easily determine whether unexpected outcomes are linked to contextual factors, adaptations made, or the intervention itself. Relatedly, the CoDDaT approach provides teams with an opportunity to carefully vet the merit of adaptations by discussing these adaptations within and across teams, reviewing associated process notes, and comparing the proposed adaptations with the outlined contextual determinants for each site before implementing them more widely. While the adaptation guidance currently available offers excellent recommendations on core elements and considerations to increase the likelihood of producing a high-quality adaptation [12], the final adaptations may be superficial, inconsequential, or detrimental to outcomes; however, this can be challenging to ascertain during the adaptation and may be better assessed during harmonization. Third, while bespoke, context-specific adaptations are worthwhile, teams may inadvertently miss some essential contextual determinants that may affect implementation and intervention efforts and outcomes. The CoDDaT framework, which considers input from wider internal and external teams, may lead to the discovery of such determinants and ensure they are all duly considered.

Caveats include the following: First, harmonized adaptations may not suit all research projects. The WHO CST intervention’s development was a global collaborative effort that minimized the influence of specific cultures and cultural norms, facilitating easier cross-cultural adaptation. The adaptation and harmonization approach may be more challenging and less successful when applied to culturally bespoke, vastly different, or very heavily adapted interventions. Deciding between minimally and heavily adapted interventions is difficult and requires navigating the tension between fidelity and fit [44]. Unfortunately, no clear criteria exist for this assessment, and researchers may need to depend on the available systematic intervention process notes, adapted materials, their own experience, and the advice of field experts to decide this. Contextual similarities that may reveal the potential for successful implementation in an alternative context can be assessed by considering: (i) similarities in the main language of communication in both contexts, as a significant component of culture is captured within the language; (ii) the prevalence of the challenge needing intervention in both contexts, as that can influence intervention strategies and levels of investment; and (iii) evidence of successful common strategies that have addressed a shared problem in both contexts, as that implies other shared challenges could be resolved using strategies found to be successful in one of the contexts.

## Conclusion

There has been a steady increase in the number of studies and projects aimed at adapting interventions for use in alternative contexts. This article shows researchers how to harmonize and update previously adapted interventions for more extensive use with the CoDDaT framework. Using the WHO CST intervention being tested within the SPARK project as a case study, it outlined the harmonization process that produced a unified version for a large-scale Randomized Controlled Trial and discussed lessons learned and implications for future research. The CoDDaT framework can inform similar projects in LMICs and high-income countries to increase research and resource efficiency, promote research collaborations across contexts, and increase the generalization of findings to larger groups. If possible, data should be collected on successful adaptation components across harmonization efforts in centralized repositories to better inform future adaptation efforts. It is, however, important to note that this approach does not discount the value of adapting interventions to suit specific cultural contexts. On the contrary, it seeks to make the adaptation process more efficient and impactful by keeping the adaptation needs to a minimum by providing access to interventions that are largely suitable to contexts based on successful implementation in similar contexts.

## Welfare of animals

This article does not contain any studies with animals performed by any of the authors.

## Guidelines

There are no applicable reporting guidelines as this work is novel.
